# Integrating DeepL Write and Claude AI to enhance argumentative writing competence of engineering students in India

**DOI:** 10.3389/frai.2026.1838463

**Published:** 2026-06-02

**Authors:** T. J. Divya, C. Alamelu

**Affiliations:** School of Social Sciences and Languages, Vellore Institute of Technology, Chennai, Tamil Nadu, India

**Keywords:** argumentative writing, Claude AI, DeepL Write, English as a Second Language, STEM education, Sustainable Development Goal 4, Toulmin argumentation model

## Abstract

**Introduction:**

Artificial intelligence (AI) tools are increasingly applied to support second language writing. However, empirical research examining their potential to enhance the argumentative writing skills of STEM engineering students, particularly in the English as a Second Language (ESL) context in India, remains limited and requires more exploration.

**Method:**

This study employed a quasi-experimental mixed-method design to investigate the effectiveness of two AI tools, DeepL Write and Claude AI, in improving argumentative writing among 40 first-year engineering students at a private university in Chennai, India. The Toulmin model of argumentation-based rubric was used to analyse the argumentative essays written by participants before and after the intervention using the AI tools. In the quantitative phase, paired-sample t-test and one-way ANOVA were employed to analyse differences in the pre-test and post-test essay scores. These results were further explained through comparative analysis of the essay scripts (n = 40) and thematic coding of participants’ semi-structured interview data (n = 23) in the qualitative phase.

**Results:**

The quantitative results demonstrate significant improvement in the AW skills of the participants (*t* = −43.72; df = 39; *p*-value < 0.001; *f* = 1.534; *p* > 0.05). The findings also indicated uniform improvements across all Toulmin components. The comparative analysis of the essays evidences the development of claims, data, warrants, backing and rebuttals, as well as improvements in linguistic features such as grammar, sentence structure and vocabulary. The thematic coding of the interview data yielded six themes related to perceived writing development, meta-cognitive awareness and the challenges and limitations of the tools.

**Discussion:**

The findings indicate the pedagogical potential of integrating DeepL Write and Claude AI to improve argumentative writing skills of Indian ESL Engineering students. The findings also highlight the effectiveness of AI tools in improving linguistic features in students’ writing. By fostering responsible and informed use of digital technology in education, this study aligns with the Sustainable Development Goal (SDG) 4 (Quality Education).

## 1 Introduction

The emergence of Generative Artificial Intelligence (GenAI) has revolutionised diverse fields and has transformed education (Wollny et al., 2021), particularly language teaching and learning. The potential of various Artificial Intelligence (AI) tools in improving language skills, listening, speaking, reading and most importantly writing skills (Bekdaş, 2026) has been explored in several research studies. Tools such as ChatGPT (Ozan and Azap, 2026; Jayakumar et al., 2025; Tayeb, 2025), Grammarly (Mekheimer, 2025), Google Translate (Alghasab, 2025) and other AI tools provide immediate personalised feedback on vocabulary, grammar, sentence structure and cohesion. Furthermore, learners perceive this feedback as accessible, fast and less judgmental than traditional instructor feedback (Mekheimer, 2025; Ozan and Azap, 2026; Mujeeb et al., 2026), which boosts confidence, promotes engagement and fosters autonomy. Therefore, AI tools are extensively utilised across diverse academic writing genres, including essays, reports, case studies, reviews and so forth, especially in tertiary-level second language contexts (Fathi and Rahimi, 2026; Bekdaş, 2026; Shahsavar et al., 2024; Alghasab, 2025).

Among various academic writing genres in which AI tools are employed, argumentative essay writing remains a complex skill as it requires not only linguistic proficiency but also the ability to organise claims, support them with evidence and address counterarguments (Nussbaum and Schraw, 2007; Chandrasoma and Jayathilake, 2022; Yang, 2022a). According to Bloom’s Taxonomy, it is a higher-order thinking skill (Anderson and Krathwohl, 2001) that promotes critical thinking, problem-solving and logical reasoning in learners (Facione, 1990; Rapanta and Iordanou, 2023; Chandrasoma and Jayathilake, 2022). Furthermore, studies indicate that it enhances communicative and persuasive language abilities (Afshar et al., 2017; Beniche et al., 2021). Argumentative Writing (AW) skills are fundamental to success in academic and professional fields across many disciplines, and its development is recognised as an important educational goal (Quintana and Correnti, 2018).

AW becomes an indispensable skill in STEM (Science, Technology, Engineering, Mathematics) education, especially in engineering fields, which require students to develop higher-order skills like problem solving, critical thinking, analytical reasoning and the ability to communicate complex ideas effectively (Barkoczi et al., 2024; Liu et al., 2025; Ahern et al., 2019; Cox and Lough, 2007; Dhanavel, 2013; Parico et al., 2020). Engineers are often expected to justify their decisions, assess alternatives and present evidence-based arguments (Müller and Hunter, 2012; Garcia and Mazzotti, 2017; Wilson-Lopez et al., 2020).

Regardless of the importance of AW skills, engineering students encounter several challenges in developing and practising it. A major obstacle is the lack of opportunities to practice and develop writing during college years, as their curriculum mainly emphasizes technical competence (Davies et al., 2021; Talib, et al., 2018). This limitation in their writing skills becomes more prominent when they write argumentative essays, which require knowledge of rhetorical structure, logical sequencing, and appropriate language use (Ferris, 1994). This challenge is intensified in ESL engineering contexts where students need significant assistance in their writing skills (Wang, 2016; Mdodana-Zide and Mafugu, 2023). Studies have identified partial understanding of the argumentation genre, problems with structuring, analysing and organising arguments and limited language proficiency as some of the reasons that lead to AW difficulty among tertiary-level second language learners (Beniche et al., 2021; Bhartia and Sehrawat, 2022; Rahmatunisa, 2014; Valero Haro et al., 2022; Ghanbari and Salari, 2022). Although studies have employed various AI tools to overcome these challenges and improve AW among ESL/EFL learners in higher education contexts, limited research has been conducted in engineering contexts, particularly in India, where learners enter higher education with heterogeneous linguistic backgrounds (Kalyanpur et al., 2022) and uneven prior exposure to formal academic writing (Harshalatha and Sreenivasulu, 2024). Given the importance of the AW among engineering students, methods to overcome the challenges and develop the skills require more empirical attention.

This study utilised a quasi-experimental mixed-method design to explore the effectiveness of two emerging AI tools, DeepL Write and Claude AI, in improving AW skills among first-year Indian ESL engineering students. This study has employed Toulmin’s model of argumentation, which deconstructs an argument into its fundamental components: claim, qualifier, grounds/data, warrant, backing and rebuttal (Toulmin, 2003) to analyse the students’ argumentative essays. By employing dual AI tools, the study seeks to evidence the effectiveness of converging multiple AI tools concurrently to improve AW.

The study addresses the following research questions:

1. Is there a significant development in first-year ESL engineering students’ argumentative writing following the use of DeepL Write and Claude AI?
2. Is there a significant difference among the Toulmin components (claim, data, warrant, backing, and rebuttal) in their levels of improvement following the intervention?
3. What are the differences in the students’ pre- and post-test essays in terms of argumentative writing components and linguistic features?
4. What are the perceptions of the students regarding DeepL Write and Claude AI in improving their argumentative writing?

## 2 Literature review

### 2.1 Argumentative writing models

There are different models that inform the teaching of argumentative essay writing, apart from structured frameworks like the Toulmin Model of Argumentation (Toulmin, 2003), including the Aristotelian argument model and the Rogerian argument model (Lunsford, 1979). The classical Aristotelian model accentuates persuasion through the integration of logical reasoning or logos, emotional appeal or pathos and credibility or ethos and is used to develop persuasive writing skills (Sichach, 2024). On the contrary, the Rogerian model is more dialogical and encourages writers to admit opposing viewpoints before presenting their position (Davis, 2012). However, the Toulmin model is suitable for analysing the internal structure of arguments in student writing, as it clearly defines elements such as claims, grounds, warrants, backing and rebuttals. This framework has been widely employed in educational research to assess and improve AW as it provides a clear, systematic method for evaluating both the content and structure of written work (Simosi, 2003; Osman and Januin, 2021). Moreover, targeted feedback based on these elements enhances learners’ ability to construct arguments (Kerman et al., 2022). Accordingly, this study defines argumentative essay writing as the capability to construct a strong written argument with a distinct claim, supported by relevant evidence and justification, while also addressing counterarguments, as reflected in the Toulmin framework.

### 2.2 Technology integrated argumentative writing

A range of digital platforms and mobile technologies have been employed to support AW development among university second language learners. Tools like EdPuzzle, Trello, Google Docs, Wordle and many more have shown to encourage goal-setting, self-monitoring, collaboration, and structured AW development (Yeganeh and Nemati, 2025; Rashtchi and Porkar, 2020). For instance, Rashtchi and Porkar (2020) found that brainstorming activity using the Wordle tool enhanced Iranian EFL learners’ cognitive skills and improved AW. Furthermore, studies have explored the impact of collaborative platforms like WhatsApp, digital games and argument maps on AW. As in the study by Haron and Kasuma (2021), which revealed that structured discussions, knowledge sharing and engagement with peers through WhatsApp enhanced the AW proficiency of Malaysian ESL university learners. Moreover, the findings of Guo et al. (2024) demonstrated that Argument Arena, an online game, improved Chinese undergraduate learners’ AW by assisting with idea generation and essay composition and by providing peer feedback. The study also reported that the platform increased interest, perceived usefulness and connectedness among learners. Additionally, Fan and Chen (2021) studied the potential of a computer-aided system (CAEWS) that creates digital argument maps to help AW of elementary students in Taiwan. The results indicated that the tool significantly enhanced their ability to structure argumentative essays compared to conventional writing methods. Collectively, findings from previous studies indicate that these digital platforms improve AW skills through peer interaction and by strengthening organisation and structuring of arguments. These tools also created increased engagement and interest among learners. Acknowledging these benefits, educators have begun to employ more innovative technology tools to improve AW skills of learners that provide immediate, personalised feedback on the grammar, clarity, coherence and cohesion of arguments.

### 2.3 AI tools to improve argumentation

Given the benefits of AI tools for second language writing improvement, there has been an increase in empirical studies that examine the potential of AI tools to assist learners in generating, evaluating and refining arguments. Research studies have examined the impact of tools such as Grammarly (Raza et al., 2024), Quillbot (Pratama et al., 2025) and ChatGPT (Li et al., 2024; Khampusaen, 2024; Yasmin et al., 2025). The study by Khampusaen (2024), for example, highlighted that the use of ChatGPT enhanced EFL university learners’ AW in terms of the development of arguments, the integration of evidence and improvement of academic tone, along with notable enhancement in their academic integrity. In addition to strengthening AW abilities, research has identified the potential of ChatGPT to reduce language errors and thereby improve learners’ overall writing skills (Yasmin et al., 2025). Furthermore, Zhang et al. (2025) investigated the impact of Spark Desk, an AI chatbot, in fostering critical thinking and intrinsic motivation in AW of Chinese EFL undergraduates. The results indicated considerable improvements in reasoning, organisation, enjoyment and reduced stress among learners following the intervention. Similarly, Guo et al. (2022) examined the effect of a chatbot named Argumate in supporting Chinese EFL students’ construction of persuasive arguments. Although the chatbot improved idea generation and counterargument construction, it is constrained by a limited database, which produces pre-defined replies rather than being generated in response to the evolving interaction.

Additionally, an increasing number of studies have employed the Toulmin Model to analyse AI-supported AW among second language learners. Though these studies adopted different methodologies, they reported a positive impact of the AI tool utilised on the development of different Toulmin components. For example, Fadilah (2026) studied the impact of ChatGPT-assisted peer collaboration on the AW of Indonesian university EFL learners. The results indicated evident improvements in warrants, backing, and rebuttals, suggesting the development of higher-order inferential reasoning in learners. At the same time, the study by Al Fraidan (2025) combined the real-time feedback offered by ChatGPT with Uncertain Motivation (UM) strategies within the Toulmin model, which improved tertiary-level Saudi EFL learners’ articulation of claims, data, backing, and rebuttals and their ability to craft structured argumentative essays. The study also found that the combination of AI and UM encouraged motivation, leading to deeper cognitive engagement in the development of the essays. Moreover, Raza et al. (2024) compared argumentative essays written with and without AI assistance among undergraduate learners. The findings showed that AI-supported essays were more aligned with Toulmin components with improved clarity of claims, logical coherence and inclusion of rebuttals. However, participants also expressed concerns like over-reliance, loss of critical thinking and ethical considerations while utilising AI writing tools.

In summary, the literature shows that AI-powered writing tools offer significant advantages for second language learners in terms of improvement in argument construction quality, logical reasoning, linguistic accuracy and cognitive ability. When combined with Toulmin’s model, these tools provide a strong framework to improve both linguistic accuracy and the logical organisation of arguments. Therefore, AI applications are being framed as valuable instructional support tools for teaching argumentation with their intelligent feedback mechanisms and reasoning frameworks.

### 2.4 DeepL Write and Claude AI

Though there is a growing body of research on the integration of AI into language instruction, particular attention must be given to emerging tools that offer advanced linguistic and cognitive support. Among these, DeepL Write (DeepL, 2025) and Claude AI (Claude, 2025) represent two increasingly relevant technologies in educational contexts. A recent development by DeepL, DeepL Write, is specifically designed to assist plurilingual users in composing texts directly in a foreign language, eliminating the translation phase, unlike DeepL Translate (Klimkowski, 2023). It also takes less time to create quality texts in the target language (formal emails, minutes or summaries). According to Chen (2023), the public beta version of DeepL Write (DeepL GmbH, Cologne, Germany), launched on January 17, 2023, suggests synonyms, completes partial phrases, and offers multiple full-sentence rewrites, all powered by contextualised examples via Natural Language Processing (NLP). It has the ability to understand context, which allows it to offer nuanced suggestions that are particularly beneficial for non-native speakers. This tool offers real-time language suggestions that help to refine sentences simultaneously while writing, making it more polished and professional by fixing errors and enhancing the coherence of texts (Ashford, 2025). It also provides alternative phrases and word choices (Klimkowski, 2023) and offers stylistic recommendations (Obari and Hiraiwa, 2024). During the drafting process, this tool assists with brainstorming, which helps to reduce difficulty in generating ideas (Ramli et al., 2024). It also minimises errors in syntax and vocabulary usage, thereby allowing the user to focus more on content development and logical argumentation. Similarly, it provides refined linguistic feedback to overcome grammatical and lexical limitations. Nevertheless, its use among students for self-assessment has raised concerns among instructors (Agrati and Beri, 2025).

Priyanshu et al. (2024) state that models, like Anthropic’s Claude, a large language model (LLM) that can comprehend and produce human-like text, have distinctive potential for rapid, effective, and scalable communication initiatives. It also helps to produce and arrange ideas by offering organised suggestions that improve the logic of the argument. Claude has the potential to hold multifaceted conversations, similar to GPT-based models. It employs the Anthropic Constitutional AI framework which trains the model for self-reflection and output adaptation based on a predetermined set of ethical principles drawn from human rights documents and public input. Claude’s high emotional intelligence enables it to respond with empathy and contextual sensitivity, which may be leveraged to scaffold AW in L2 contexts, particularly with regard to affective variables such as writing anxiety and motivation, associated with learners’ performance (Anthropic, 2025). Claude AI is being recognised as a writing assistant in higher education that helps with generating ideas and summarising content (Amelia et al., 2024). When used with DeepL Write, Claude AI organises the overall structure and coherence of written texts. Initial drafts of students’ essays may lack coherent organisation; however, AI-driven revisions using tools such as Claude AI can help restructure the content to improve clarity and persuasiveness. This “dual-tool” strategy is particularly useful in AW, where the linguistic accuracy is as important as the structuring of ideas.

## 3 Research gap

The constructive impacts of integrating various AI tools to enhance AW skills among tertiary-level Asian EFL and ESL learners are enumerated in the previous studies (e.g., Zainuddin and Rafik-Galea, 2016; Osman and Januin, 2021; Yang, 2022a; Al Fraidan, 2025). Various dimensions, including the changes in the articulation of arguments, students’ perceptions regarding the AI tools, quality of feedback provided by the tools and linguistic improvements are highlighted in these studies.

- However, limited studies have explored the combined potential of DeepL Write and Claude AI to improve AW.
- Additionally, the impact of AI tools to improve AW among engineering students in India remain underexplored.

Indian higher education is characterised by large class sizes, limited opportunities for personalised feedback, and different English proficiency levels among students. These contextual factors necessitate interventions that are scalable, and capable of supporting individual learning needs. Consequently, there is a need for research exploring the potential of these tools to bridge instructional gaps in writing pedagogy, particularly in contexts where real-time, individualised support is scarce. Focusing specifically on the needs of ESL Engineering students in India, the research addresses this gap and indicates the need to incorporate AI tools into the curriculum to foster AW skills.

## 4 Methodology

### 4.1 Research design

**Figure 1:**
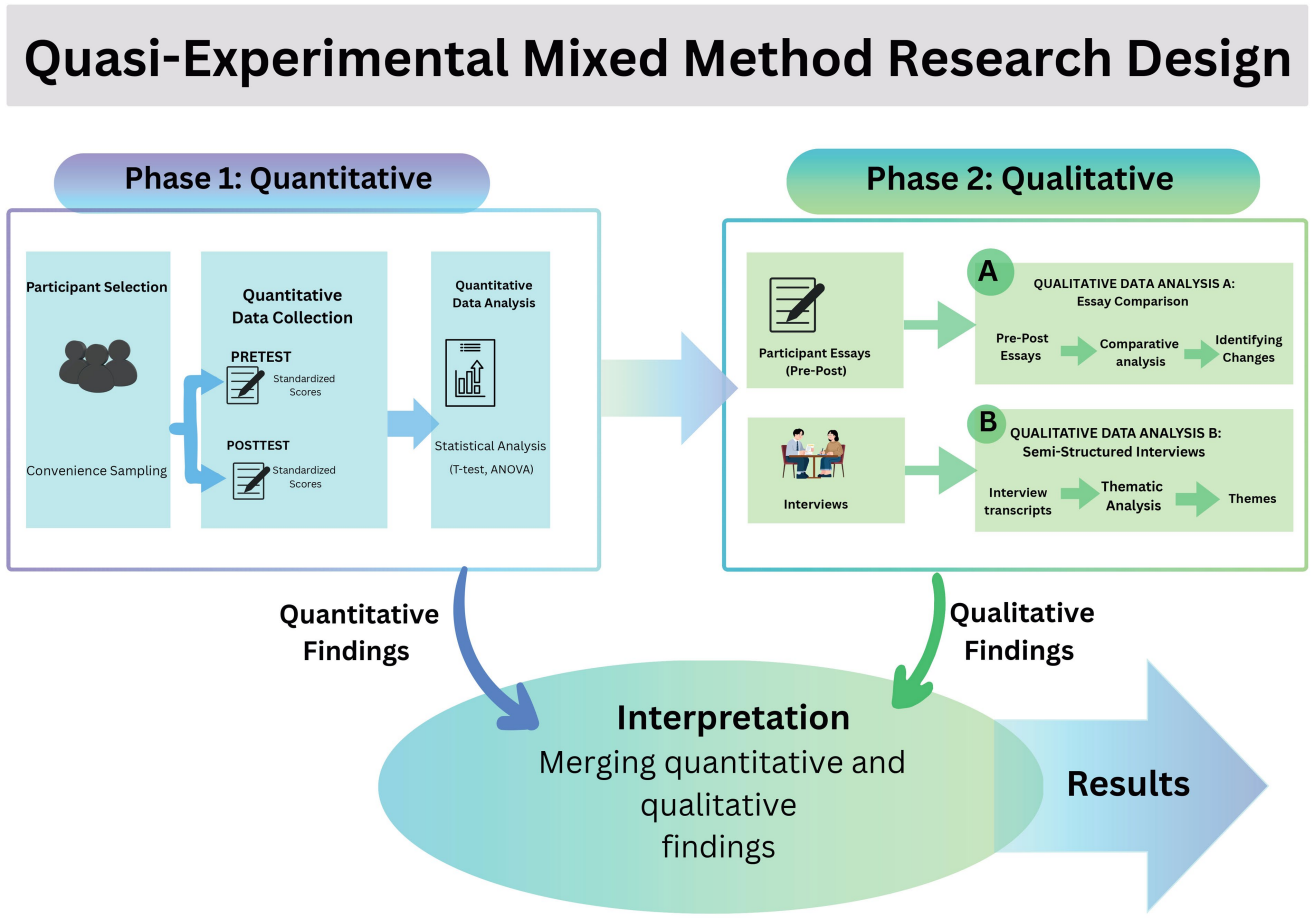
Research framework.

The study adopted a quasi-experimental mixed-method research design to investigate the effectiveness of Claude AI and DeepL Write to develop argumentative writing skills among ESL Engineering students. It has two distinct phases; the first phase involved collecting and analysing quantitative data, followed by the second qualitative phase substantiating the results of the previous phase (Creswell and Clark, 2017). The research design and study procedure is visually represented in Figure 1. The study data were collected from a single group, which involved measuring the same group before and after the intervention (Pole, 2007; Creswell and Creswell, 2018). Due to practical constraints in the classroom setting, students from one class were selected using convenience sampling without random assignment. Therefore, the research used a single-group design, combining quantitative measures of writing performance with qualitative inputs from comparative analysis and student interviews. Creswell and Creswell (2018) note that this design is appropriate in contexts where random assignment and control groups are not feasible. In the absence of a control group, efforts were taken to ensure internal consistency by conducting the treatment within a limited time frame, by applying consistent testing procedures and by using the same rubric for both tests. Consequently, this design was considered appropriate to achieve the research objectives under the existing conditions.

### 4.2 Participant demographics

The study’s participants comprised of 40 first-year Engineering students enrolled in a private university in Chennai, India, selected through convenience sampling from an English course. The primary language (L1) of the participants included Tamil, Malayalam, Telugu and Hindi. Based on their L2 proficiency assessed by the university-administered English proficiency test at the beginning of the semester, students were assigned to this course. The sample was taken from students with average scores in test, which indicates their similarity in English proficiency levels. The average age range of the participants that included 12 females and 28 males, was 18 to 20 years. The participants were informed about the study objectives before data collection, and written consent to participate was collected from each of them. The study also received approval from institutional ethical review board.

### 4.3 Procedure

The study was conducted over five consecutive days to ensure adequate time for each phase and reflection. It consisted of three main phases: pre-test, intervention, and post-test, followed by qualitative data collection.

**Figure 2:**
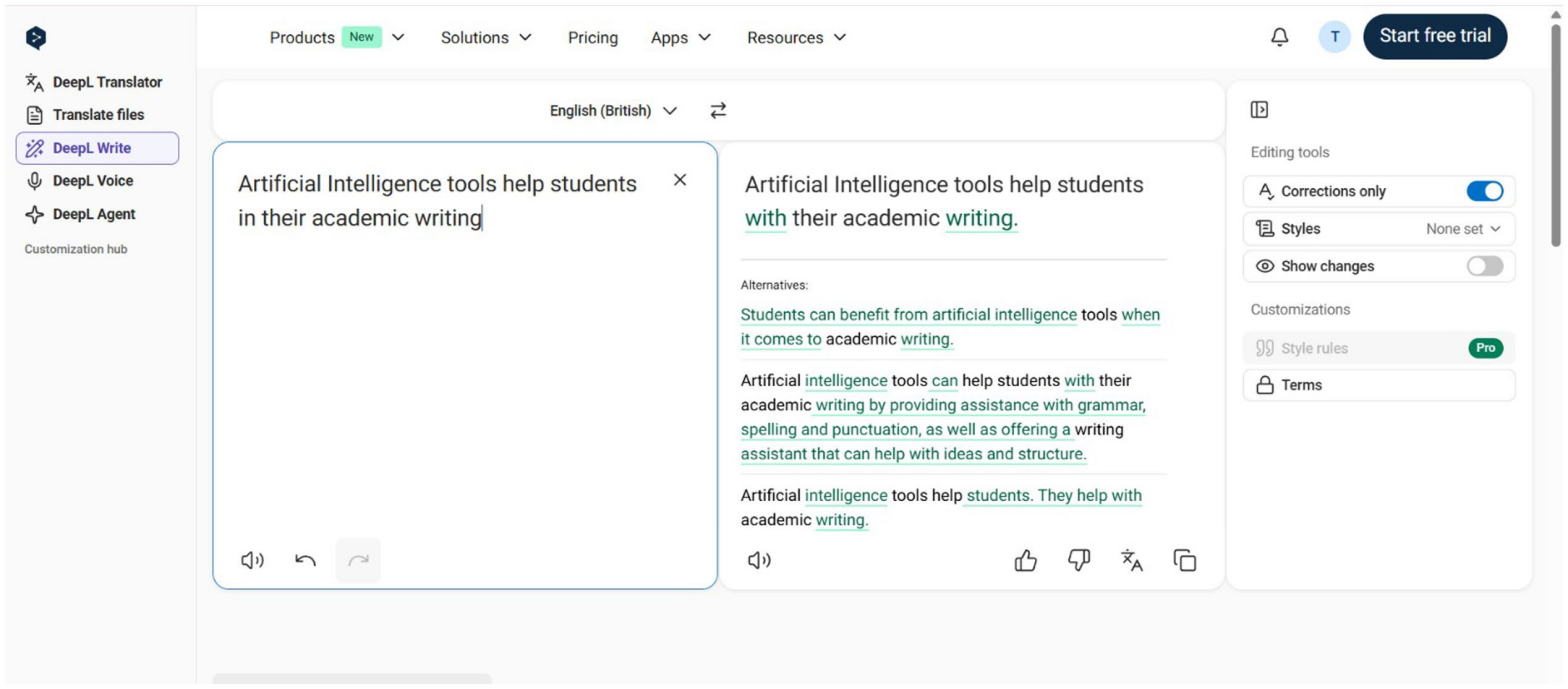
Interface of DeepL Write.

**Figure 3:**
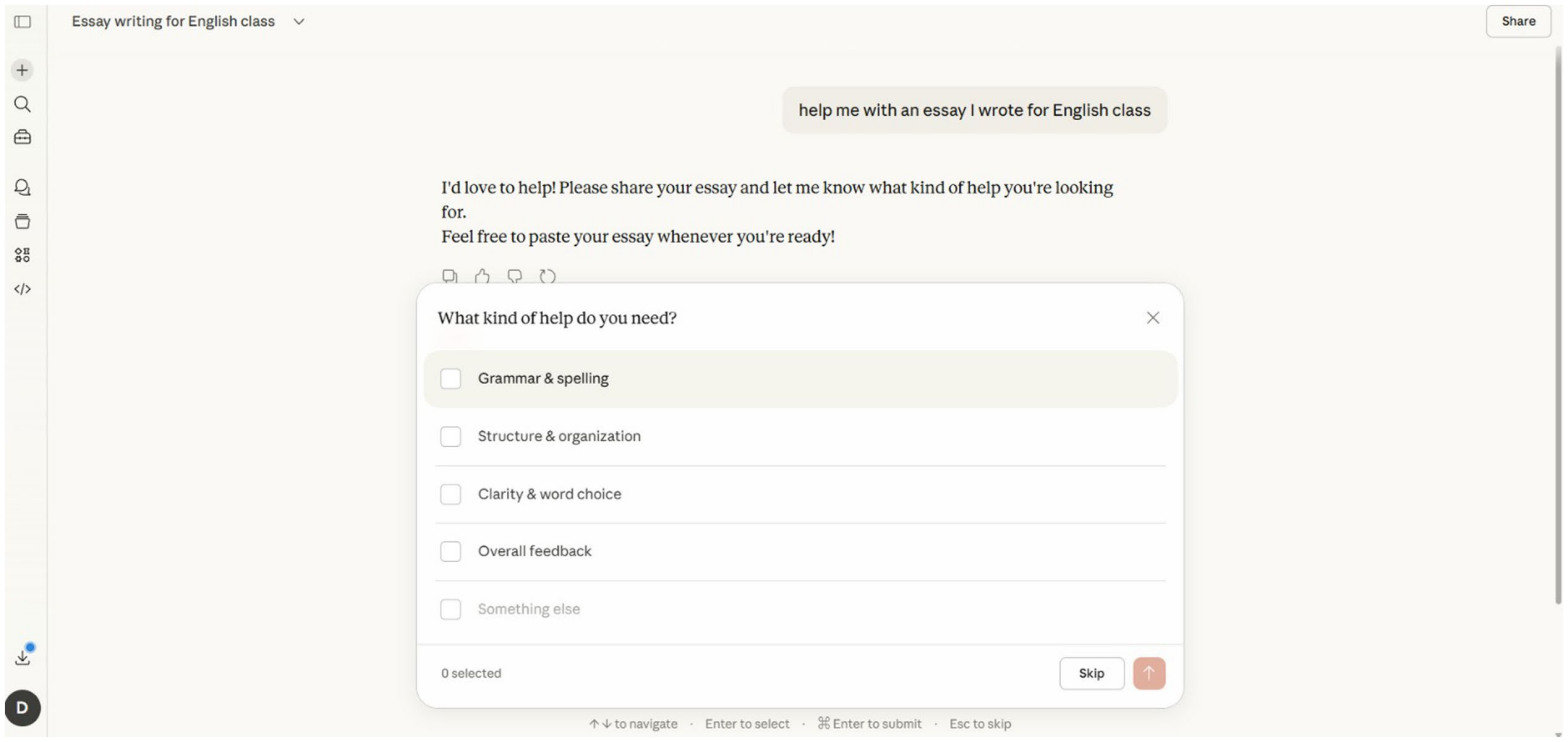
Interface of Claude AI.

- On Day 1, participants completed a pre-test in which they independently wrote an argumentative essay on the topic ‘Will Artificial Intelligence designs replace human engineers in the future?’ provided to them, without using any digital or AI tools, to establish a baseline for their writing proficiency. The pre-test session took roughly 1 h. Students were expected to write essays of approximately 300–400 words for both pre- and post-tests.
- The intervention phase began on Day 2, with a 2-h structured training session that introduced all the students to DeepL Write and Claude AI (Sonnet 4.5 version). During the session, explicit instruction was provided on utilising DeepL Write to improve the grammar and vocabulary of the essay they wrote (Figure 2) (DeepL, 2025), followed by instruction on using Claude AI for revision to improve logical sequencing of ideas, coherence and essay structure (Figure 3) (Claude, 2025).
- On Days 3 and 4, there was a practice session for 2 h each, where students used AI tools to improve the argumentative essays they wrote on different essay topics. Students wrote the essays independently in a quiet computer lab, with internet access required for using the AI tools.
- The post-test phase (1 h) occurred on Day 5, where participants wrote a second argumentative essay based on the same topic given for the pre-test. So as to minimize external assistance, distractions, and to maintain consistency, the essays were written in a supervised independent setting without any AI assistance.
- Finally, an interview occurred (2 h) on Day 5, which included interviews with 23 students to gather qualitative data on student perceptions of writing with AI, conducted on a one-to-one basis with an approximate duration of 10 min per interview session.

Students were trained throughout the intervention to avoid using AI-generated content. They were provided with guidelines to use the AI tools only for brainstorming and revising their work, and with instructions to critically review the suggested changes while keeping overall control of their writing. This collaborative use of AI was emphasized through instructions and monitored during interviews to ensure that AI was used to support learner-driven improvement, not to replace authorship.

### 4.4 Data collection and analysis

#### 4.4.1 Quantitative phase

**Figure 4:**
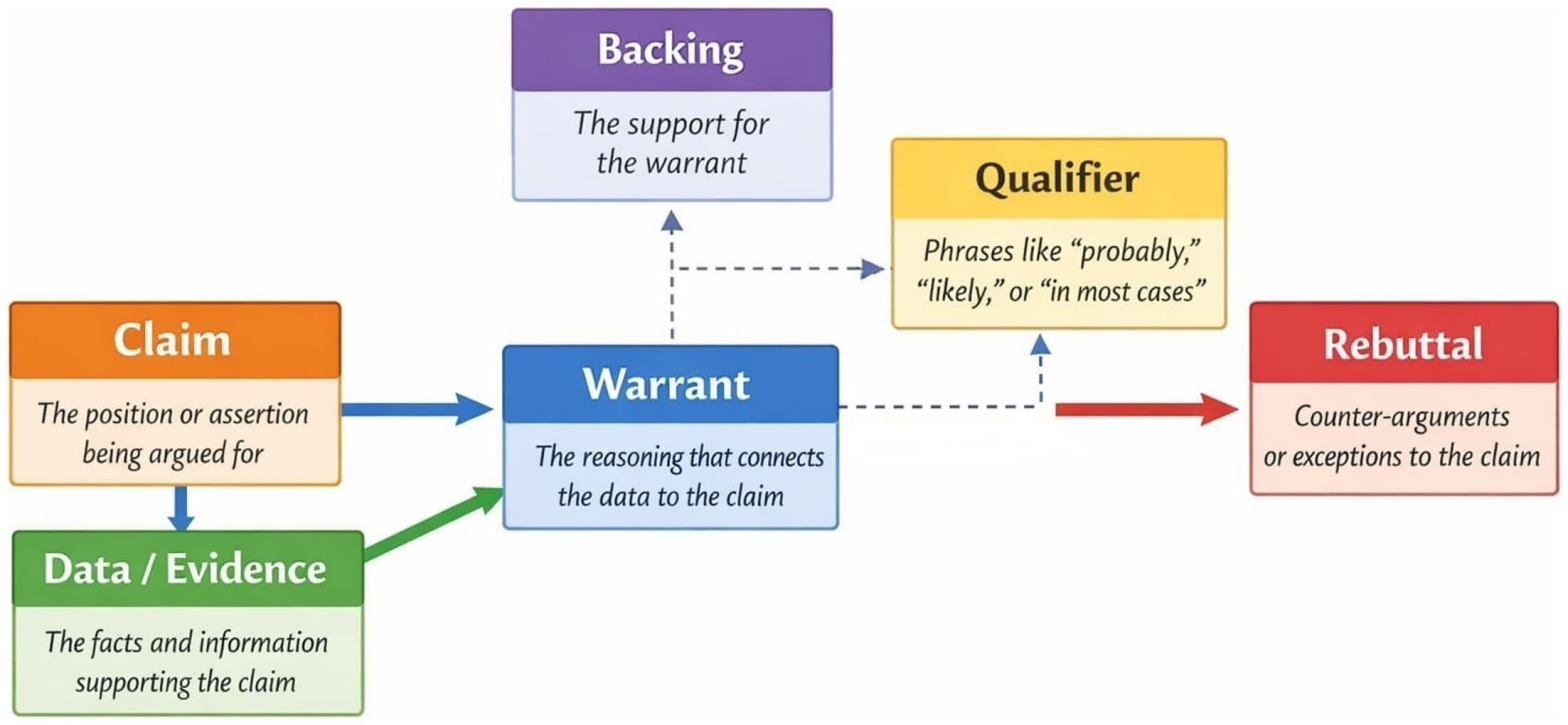
Toulmin argumentation model (Toulmin, 2003).

The rubric to assess of the pre- and post-test essays was based on Toulmin’s model of argumentation (Toulmin, 2003) and was adapted from Yang (2022b). It evaluated five elements in the Toulmin argumentation model: claim, grounds, warrant, backing, and rebuttal. Each element was scored on a scale of 0 to 3, giving a total score of 15. Figure 4 shows the model, and Table 1 explains each element. The scoring was conducted separately by two raters with advanced degrees in applied linguistics who have previously assessed argumentative writing in ESL contexts.

**Table 1:** Definitions of the six elements of Toulmin’s argumentation model.

Six elements of Toulmin’s argumentation model	
Element	Definition
Claim	It refers to the conclusion to be argued (Toulmin, 2003).
Data	It refers to the specific facts relied on to support a given claim (Toulmin, 2003).
Warrant	It serves as the bridge to justify how the claim is derived from the data (Toulmin, 2003).
Backing	It refers to facts, authorities, or explanations used to strengthen or support the warrant (Toulmin, 2003).
Qualifier	Qualifiers are modals, such as probably, possibly, perhaps (Toulmin, 2003).
Rebuttal	It specifies the conditions which might defeat the major claim (Toulmin, 2003).

##### 4.4.1.1 Reliability and validity

In order to ensure reliability, the raters were trained on the rubric elements before scoring to ensure consistent application. Inter-rater reliability was computed using ICC 3, yielding a coefficient of 0.87, indicating a high level of agreement. Descriptive and inferential statistical analyses of the pre-test and post-test scores were performed on SPSS software.

A paired-samples t-test was considered appropriate to examine statistically significant differences between the mean pre-post-test essay scores or within-group analysis. To identify which among the Toulmin components showed the most significant improvement, one-way ANOVA followed by *post hoc* Tukey HSD tests was performed on post-test scores for each component. ANOVA is suitable for comparing means across more than two groups or between-group comparisons. Prior to these parametric tests, assumptions of normality and homogeneity of variance were verified using the Shapiro–Wilk (*p* > 0.588) and Levene’s tests (*p* > 0.181). The results (*p* > 0.05) satisfied these assumptions, validating the use of these tests for subsequent comparisons.

#### 4.4.2 Qualitative phase

##### 4.4.2.1 Comparison of pre-test and post-test essays

The pre-test and post-test essay drafts of the participants were compared to find the differences in the construction of claim, grounds, warrant, backing, and rebuttal. Additionally, the essays were analysed to identify the development of linguistic features like grammar, sentence structure and vocabulary. This analysis explained the difference in the scores and strengthened the credibility of the findings.

##### 4.4.2.2 Semi-structured interviews

To gather students’ perspectives, semi-structured interviews were conducted in-person with a subset of 23 participants who provided consent to participate. The interview protocol comprised of two main open-ended questions that focused on learners’ perceptions of their AW improvement using the two AI tools. Follow-up questions were also asked to gather detailed responses and clarify student experiences. For the subsequent transcription and analysis, all interviews were audio recorded. The responses were transcribed and thematically analysed independently by the researcher following Braun and Clarke (2006) six steps framework for thematic analysis. The coding was performed using a trial version of MAXQDA Analytics Pro 24. This mixed-method design provided a holistic view of the intervention’s impact, combining measurable writing outcomes with learner experiences.

## 5 Findings

### 5.1 Descriptive statistics

**Figure 5:**
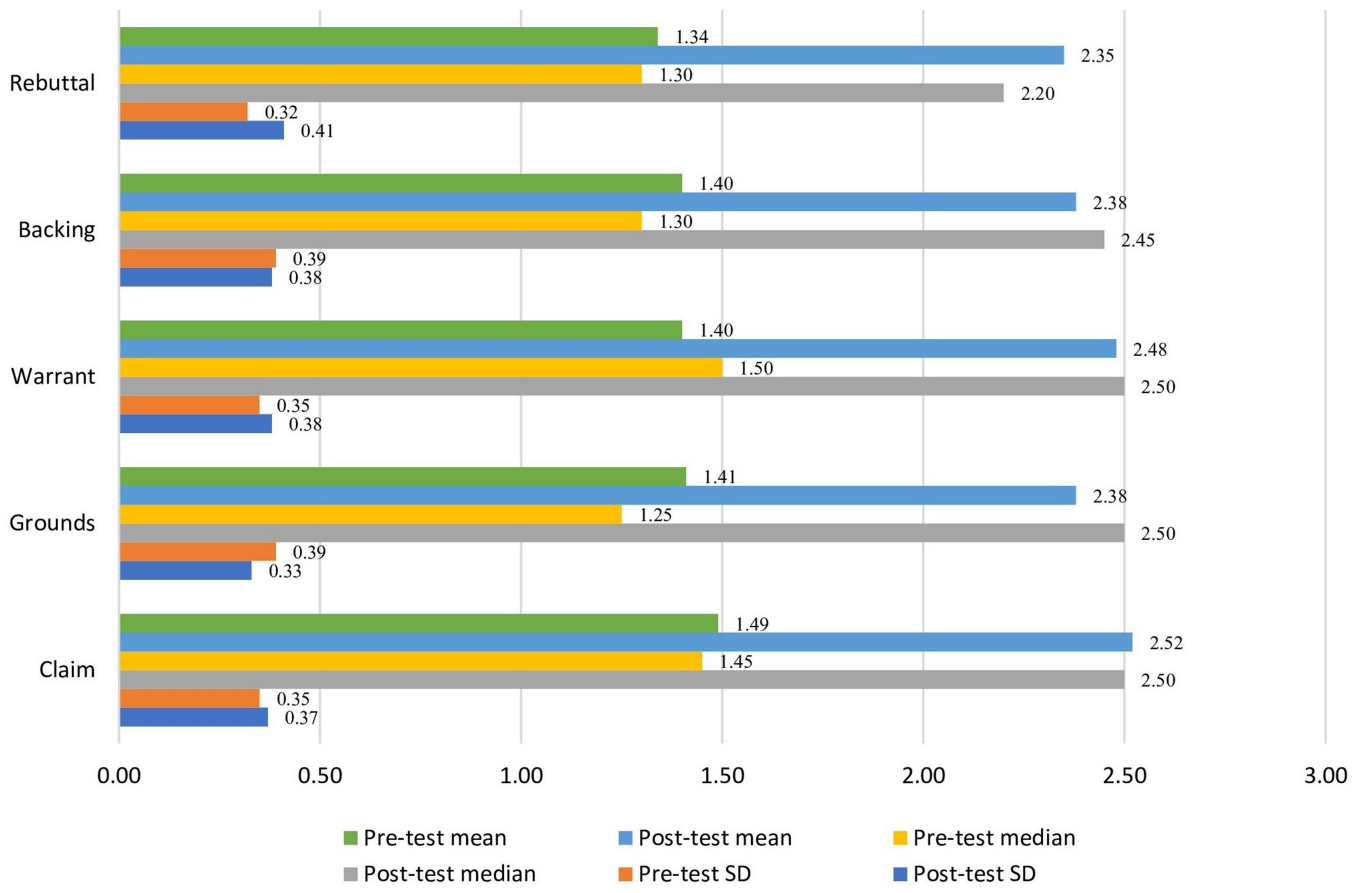
Descriptive statistics of essay scores.

Figure 5 presents the descriptive statistical analysis of pre-test and post-test scores for the five components of the Toulmin model. Each component, evaluated in terms of mean, median and standard deviation (SD), shows an upward trend indicating an overall enhancement in the participants’ AW skills following the intervention.

### 5.2 Inferential statistics

The paired-samples t-test (Tables 2, 3) showed statistically significant improvement in students’ AW scores following the AI tool intervention. The mean pretest score was 7.05, which increased to 12.13 in the post-test, giving a mean difference of −5.09. The t-value of −43.72 (df = 39) and the *p*-value of 0.000 indicate a highly significant result at the 95% confidence interval, suggesting that the improvement was not due to chance. These findings demonstrate that the integration of DeepL Write and Claude AI had a meaningful impact on enhancing students’ ability to construct coherent, logically structured argumentative essays.

**Table 2:** Paired samples *t*-test results.

Paired samples statistics				
AW	Test score	Mean	*N*	SD
Before and after intervention	Pre total (15)	7.05	40	0.51
	Post total (15)	12.13	40	0.75

Paired samples test					
AW	Comparison	Paired differences		*t*	Sig.
		Mean	SD		
Before and after intervention	Pre total (15) – post total (15)	−5.09	0.74	−43.72	0.000

**Table 3:** Paired samples *t*-test results across Toulmin components.

Paired samples test					
Sources of variance	Comparison	Paired differences		*t*	Sig.
		Mean	SD		
Pair 1	Pre claim – post claim	−1.03	0.39	−16.555	0.000
Pair 2	Pre grounds – post grounds	−0.98	0.38	−16.073	0.000
Pair 3	Pre warrant – post warrant	−1.08	0.38	−17.924	0.000
Pair 4	Pre backing – post backing	−0.99	0.51	−12.271	0.000
Pair 5	Pre rebuttal – post rebuttal	−1.01	0.41	−15.754	0.000

A one-way ANOVA (Table 4) and Tukey HSD *post hoc* test (Table 5) were performed to evaluate the pairwise differences in marks across the different AW components (Claim, Grounds, Warrant, Backing, and Rebuttal). The analysis revealed no significant differences in mean post-test scores across the five components at 95% confidence (*p* > 0.05). The homogeneity of means across groups underscores the equitable impact of the AI-mediated instruction, implying that the tools facilitated balanced development across all argumentative sub-skills rather than improving any particular component. This finding relates to the pedagogical objective of enhancing integrated argumentative competence rather than acquiring each sub-skill in isolation.

**Table 4:** One-way ANOVA of AW components.

Groups	Sum of squares	df	Mean square	*F*	Sig.
Between groups	0.890	4	0.223	1.534	0.194
Within groups	28.299	195	0.145		
Total	29.190	199			

**Table 5:** Multiple comparisons of AW components using Tukey HSD.

(I) AW component	(J) AW components	Mean difference (I-J)	Sig.
Claim	Grounds	0.1425	0.453
	Warrant	0.0450	0.984
	Backing	0.1425	0.453
	Rebuttal	0.1750	0.244
Grounds	Claim	−0.1425	0.453
	Warrant	−0.0975	0.783
	Backing	0.0000	1.000
	Rebuttal	0.0325	0.995
Warrant	Claim	−0.0450	0.984
	Grounds	0.0975	0.783
	Backing	0.0975	0.783
	Rebuttal	0.1300	0.547
Backing	Claim	−0.1425	0.453
	Grounds	0.0000	1.000
	Warrant	−0.0975	0.783
	Rebuttal	0.0325	0.995
Rebuttal	Claim	−0.1750	0.244
	Grounds	−0.0325	0.995
	Warrant	−0.1300	0.547
	Backing	−0.0325	0.995

### 5.3 Comparative analysis

The comparative analysis of the pre-test and post-test essays shows significant improvement in participants’ AW skills and language use following the use of the two AI tools. There is an evident change in the way the learners construct arguments before and after the intervention.

#### 5.3.1 Development of argumentative writing across Toulmin components

To examine qualitative changes in participants’ argumentative writing, the pre-test and post-test essays of respondents were compared across the AW features, which substantiate the improvement in the post-test scores. This analysis showed how students’ AW evolved in clarity, structure and reasoning following the use of the AI tools. The changes in the Toulmin components across the pre- and post-essays of participant 35 are discussed below.

##### 5.3.1.1 Improvement in claim clarity

The claims written by the participant in the pre-test were generally simple and unsupported, it was written as standalone sentences, typically supported by minimal reasoning. The post-test response demonstrated more precision and balance by limiting overgeneralisation and acknowledging exceptions.

Pretest: “AI might take engineering jobs someday.”

Post-test: “AI may replace some engineering tasks in the future, but it is unlikely to replace engineers completely.”

##### 5.3.1.2 Increased use of supporting data

The claim was followed by a vague supporting idea in the pre-test, which was more of a personal opinion than grounds. The intervention improved the framing of the grounds by making it more detailed, specific and relevant to the claim.

Pretest: “AI programs can design models and solve problems faster than humans.”

Post-test: “AI systems can assist with parts of engineering design and analysis, but key decisions still rely on human judgment.”

##### 5.3.1.3 Emergence of warrants and backing

An important difference between pre- and post-test essays was the presence of a warrant. Though the students did not learn to write them explicitly, the changes were noticeable. In the pre-test, warrants were largely absent making the logical connection between claim and ground implicit. However, in the post-test, students began to express underlying assumptions more clearly, showing an improvement in awareness about linking claim and grounds.

Post-test: “Even though AI can solve problems fast, it cannot handle complex decisions that need human judgment and experience.”

Backing for these warrants also showed improvement.

Post-test: “Engineering projects involve safety standards and ethical responsibility, which require human oversight.”

##### 5.3.1.4 Inclusion of rebuttals

The framing of rebuttal is another major improvement, which was absent in some of the pre-test essays or presented as a weak alternative perspective. In contrast, in the post-test essays, students began to incorporate rebuttals that recognised opposing views while maintaining their original stance. This change indicates the development in the students’ awareness about including alternative viewpoints in their argumentative essays.

Pretest: “Engineers can use AI to improve their work.”

Post-test: “Although some believe AI will replace engineers, engineers are still needed to supervise systems and take responsibility for outcomes.”

Collectively, the findings indicate a shift from largely opinion-based responses in the pre-test to more structured and coherent arguments in the post-test. While certain elements, particularly the structuring of warrants, remained implicit, the post-test essays exhibited clearer organisation, more developed support, and improvement in presenting rebuttal, suggesting growth in students’ argumentative writing competence.

#### 5.3.2 Linguistic improvements in writing

Compared with the pre-test essays, students’ post-test essays showed measurable developments in terms of linguistic features like tense consistency and subject-verb agreement, sentence structure and vocabulary.

##### 5.3.2.1 Tense consistency

The post-test essay samples indicated noticeable development in the tense consistency within a sentence compared to the pre-test samples. It moved from the present tense (analyse) to the past tense (helped) within the sentence, which disrupts the tense consistency. But the post-test essay of the same student had no such inconsistencies.

Pretest (participant 4): “AI systems analyse data and helped engineers make better designs.”

Post-test (participant 4): “Many engineers use AI-based software to reduce routine workload and save time.”

##### 5.3.2.2 Subject-verb agreement

Improvements are also identifiable in subject-verb agreement. The pre-test samples had minor subject-verb disagreement, where the plural verb ‘improve’ does not agree with the singular subject ‘use’. The post-intervention essay by the same participant displayed good subject-verb agreement.

Pretest (participant 16): “The use of AI tools improve the quality of engineering work.”

Post-test (participant 16): “The integration of AI systems allows engineers to manage complex processes more effectively.”

##### 5.3.2.3 Sentence structure

Regarding sentence structure, pretest samples had poorly connected clauses, While in the post-test there was improvement in the clause connection, which reflects increased control over sentence construction.

Pretest (participant 40): “AI is useful it can do things better.”

Post-test (participant 40): “Engineers use AI because it makes their work easier.”

##### 5.3.2.4 Vocabulary choices

The post-test essays also showed improvement in vocabulary choices. For example, the post-test essays included terms like ‘analyse’, ‘enhance’, ‘efficiency’, ‘simulation’, and so on that were absent in the pre-test samples. The language-related themes identified in the interview data are substantiated by changes in the students’ pre- and post-test essays, indicating alignment between students’ perceptions and their written performance.

### 5.4 Participants’ perceptions of the two AI tools

The thematic analysis of the interview transcripts identified 6 themes; Language Accuracy and Fluency, Writing Ease and Efficiency, Idea Generation and Content Development, Writing Style and Professionalism, Meta-Cognitive Awareness and Learning, Challenges and Limitations. The initial coding obtained 17 subthemes, which were then consolidated into the six primary themes. The themes, subthemes and sample excerpt from participant responses are given in Table 6.

**Table 6:** Thematic analysis.

Theme	Sub-themes	Example quote
Language accuracy and fluency	Grammar correction and sentence structure	“DeepL Write and Claude AI gave me an insight on how to frame sentences properly and avoid grammatical errors.”
	Vocabulary development	“Better terms for writing and increased vocabulary.”
Writing ease and efficiency	Clarity and tone	“DeepL Write helps improve my grammar, sentence clarity, and fluency.”
	Ease of composing	“The process of writing is made simple by using AI tools.”
	Time-saving	“DeepL polishes my text… saving me time and effort.”
	Confidence and motivation	“Now my skills have improved to a point where I write essays at ease…”
Idea generation and content development	Brainstorming and idea support	“Claude AI helps with brainstorming, structuring arguments, and generating well-reasoned content.”
	Organising thoughts	“I had learnt the way to describe my thoughts in the correct format.”
	Argumentation	“Improved argumentation style and idea generation.”
Writing style and professionalism	Stylistic improvement	“Writing style has greatly improved along with choice of words.”
	Professional tone	“The writing is more polished. It helps make my casual writing style into a professional one.”
	Coherence and refinement	“Instead of a randomly ordered sentence, I am able to make my point clear, crisp, and elegant.”
Meta-cognitive awareness and learning	Learning through correction and feedback	“It is correcting the sentences and creating the good look for the sentences.”
	Structuring and reflective awareness	“I had learnt the way to describe my thoughts in the correct format.”
Challenges and limitations	Cognitive and expression challenges	“The suggestions sound too formal or unnatural, requiring manual adjustments, occasionally oversimplifying ideas. Additionally, relying too much on AI can reduce originality and personal writing style.”
	Functional and technical constraints	“The tools struggle with deeply nuanced arguments” “DeepL Write has a word limit.” “Confusing output, or the answers aren’t perfect.”
	Refinement and usability needs	“Requires manual refinement to ensure accuracy and originality.”

## 6 Discussion

The present study investigated the combined impact of DeepL Write and Claude AI on AW among first-year ESL Engineering students in India. The findings from the statistical analyses demonstrated a considerable enhancement in overall AW performance along with equal improvements across individual argumentative components following the AI-assisted intervention. These findings were validated through a comparative analysis of the pre-test and post-test essays and a thematic analysis of participants’ interview transcripts. The findings suggest that employing AI tools scaffolds both persuasive and linguistic aspects of argumentation.

The aim of the first research question was to determine whether there was a measurable improvement in AW skills of the participants following the intervention. The statistical analysis results indicated significant differences in post-intervention scores, suggesting that the participants’ AW skills improved after using the two AI tools. The second research question was to assess whether the improvement varied across the five components of the Toulmin model, that is, Claim, Grounds, Warrant, Backing, and Rebuttal. The analysis of post-test scores indicated no statistical differences between these components, suggesting participants’ writing improved equally across the five components after the intervention. This suggests that the improvement in the participants’ capacity to construct well-structured arguments was balanced, indicating the additional efficacy of AI-supported instruction. These findings reinforce previous research by Pratama et al. (2025), Li et al. (2024), Khampusaen (2024), Darmawansah et al. (2024) and Guo et al. (2022), which indicates the capacity of AI-assisted writing tools in improving the quality of argumentative essays by university EFL learners.

Additionally, the comparative analysis of the pre-test and post-test essays demonstrates improvements in AW and linguistic features, answering the third research question. The comparison showed that the post-intervention essays improved in terms of articulation of all five Toulmin components, validating the changes in students’ scores. In the post-test drafts, the claims written by the students became more specific, the grounds were relevant to these claims, and the rebuttals were framed with greater relevance to the claims. Importantly, the students demonstrated better awareness in the construction of warrants and backing. This is consistent with the findings from previous studies that demonstrated improvements in the construction of claims, grounds, warrants, backing and structuring of rebuttals following the utilisation of different AI tools (Fadilah, 2026; Guo et al., 2022; Raza et al., 2024; Al Fraidan, 2025). For instance, the findings support the outcomes reported by Guo et al. (2022), who found that Argumate, an AI chatbot, improved the integration of counterarguments in argumentative essays of EFL students. Additionally, the results by Al Fraidan (2025) demonstrated development in the construction of argumentation components among EFL learners’ AW following the use of ChatGPT. Thus, in line with previous studies, the findings suggest that AI tools, when integrated into instructional practice, can support the improvement of key components of students’ AW. The findings, however, contradict those of Stofiana et al. (2025) who found that though students used AI to support their AW, their rebuttal construction remained poorly developed compared to claims, data and warrants.

The analysis of the essays further identified language improvements, like increased accuracy in tense usage, subject-verb agreement, sentence structure and more refined vocabulary choices. The pre-test essays had inconsistent use of tense within sentences, incorrect pairing of singular subject with plural verb, limited connection between clauses and inadequate range of vocabulary choices. However, the post-test essays improved across all these features, indicating the potential of the tools to improve the linguistic abilities of ESL learners. The findings correspond with the results of previous studies, which have shown that employing AI writing tools improves grammar, vocabulary and writing competence (Syafei and Nuraeningsih, 2025; Kayal, 2024; Cai, 2026). Similar findings were reported by Zhao (2025), who found that AI tools improved linguistic precision in EFL learners’ writing tasks by modifying sentence structure, in addition to grammar and vocabulary. The findings are also consistent with previous research, which reported that AI feedback significantly improved ESL university learners’ academic writing by enhancing consistency in the tense usage and reducing errors (Mujeeb et al., 2026).

Student perceptions from the interviews report the effectiveness of these AI tools and answer the fourth research question. All respondents reported a perceived improvement in their ability to construct arguments. This aligns with the finding that real-time feedback improves learner autonomy and promotes writing coherence (Nussbaum and Schraw, 2007). They also indicated improvements in language accuracy and fluency, especially in grammar correction, vocabulary use and sentence clarity, which align with the language improvements identified in comparative analysis.

Alongside surface-level language corrections, respondents highlighted improvements in writing ease and efficiency, which positively impacted their motivation. Participants reported that the tools made writing easier, saved time and boosted their confidence. This finding supports previous research on the role of AI in enhancing writing skills and motivation. For instance, Song and Song (2023) found that ChatGPT helped in developing the academic writing skills and motivation among EFL students. Several participants also reported that their interactions with AI-based feedback led to better awareness of coherence, tone, and clarity, consistent with the findings by Mekheimer (2025). Both tools also supported learners with generating ideas and developing content, helping learners brainstorm, organise and structure arguments. The conventional ESL writing instruction often underemphasises these aspects of writing, however, structured interactions with the AI tools enhanced learners’ cognisance of them.

Moreover, enhancements in writing style and professionalism were also noted, with participants describing how AI suggestions refined their expression. The AI tools exposed learners to advanced academic registers changing their writing style. Apart from these benefits, some responses indicated meta-cognitive awareness, like reflection on sentence structure and self-correction, highlighting the pedagogical potential of the tools. This perception supports findings from previous research that indicate the supporting role of AI tools in improving metacognitive abilities in students (Yang and Xia, 2023). Taken together, the findings of the present study reinforce previous research demonstrating that AI writing tools can support improvements in both organisation of arguments and linguistic accuracy.

However, the interviews also revealed challenges related to the use of the AI tools. Of the 23 participants, 11 reported challenges and limitations that they experienced while using the tools. This included cognitive and expression challenges, like the fear of over-reliance on AI, which might inhibit their ability to think independently and apply their writing capabilities outside digital environments. Similar learner concern about over-dependence on AI tools was expressed in previous studies by Mujeeb et al. (2026) and Raza et al. (2024). Additionally, systematic and narrative reviews provide broader evidence to the apprehension that overdependence on AI can affect essential cognitive abilities (Zhai et al., 2024) and reduce learner autonomy in writing (Akanda and Talukder, 2026). They also reported character limits in DeepL Write and verbosity in Claude AI. Participants also reported that AI-generated feedback was occasionally overly general or formal, insufficiently tailored to the specific content produced by them. They also reported refinement and usability needs, which necessitated manual adjustments to align with the intended meaning. These challenges affected learners’ experience with the writing tools to some extent. These observations suggest that, though writing with AI is useful, educators should guide learners in its use, encouraging critical reflection and independent judgment rather than supporting the unquestioned acceptance of AI-generated content.

This study highlights both the affordances and the constraints of AI-assisted writing and demonstrates how structured AI integration can support learner autonomy, academic literacy, and lifelong learning skills. It makes a distinction between using AI as a collaborative support and AI-generated writing. This is important in addressing concerns in the computerised writing assessment literature regarding issues of authenticity and authorship of the text (Shermis and Burstein, 2013). Thus, it aligns with the study by Coman et al. (2026), which advocates the need to maintain academic integrity while employing AI tools for various writing tasks. It contributes to the understanding of the use of AI tools in academic contexts and offers insights to its sustainable and responsible integration in education, consistent with SDG 4 (Quality Education), particularly targets 4.4 and 4. a by promoting inclusive, effective, and technology-enhanced learning opportunities in higher education (United Nations, 2015).

## 7 Conclusion

This study highlights the collaborative role of two AI writing tools, DeepL Write and Claude AI, to improve the AW skills of Indian ESL Engineering students within STEM education. The quantitative findings indicate notable improvements in participants’ AW, in terms of all five Toulmin components, namely claim, grounds, warrant, backing and rebuttals. These findings were corroborated by the comparative analysis, indicating corresponding changes in students’ written performance. The analysis identified improvements in the structuring of the five components of AW and development of linguistic features like grammar, sentence structure and vocabulary. Additionally, the thematic analysis of student interviews identified six themes, Language Accuracy and Fluency, Writing Ease and Efficiency, Idea Generation and Content Development, Writing Style and Professionalism, Meta-Cognitive Awareness and Learning and Challenges and Limitations, which are related to developments in language accuracy as well as challenges encountered while using the AI tools. A notable finding of this research is that students are able to critically engage with AI-generated suggestions as opposed to accepting them passively. The tools, DeepL Write and the Claude AI provide scaffolding for AW by equipping the participants to refine both content and language in real time, while maintaining academic integrity as they help users in modifying their own content. This study demonstrates that the strategic application of AI tools can improve both organisational quality and linguistic accuracy of AW. This fusion of technology and pedagogy presents a promising direction for future research and instructional practice within STEM education. The findings of this study contribute to SDG 4 (Quality Education) (United Nations, 2015) by equipping students with responsible use of technology for education. By demonstrating how structured AI integration can support learner autonomy, academic writing, and lifelong learning skills in higher education, the study proves that AI represent a powerful pedagogical frontier in ESL writing instruction.

## 8 Limitations and future directions

Although this study’s findings contributed many insights regarding the applications of AI tools in AW and related improvements in the second language of the participants, it is important to acknowledge the several limitations of this study while interpreting the results. Most importantly, the study used a relatively small sample collected from an institution in Chennai, Tamil Nadu, and was conducted over a short intervention period which might hinder the generalisability of the findings. Further research should investigate the long-term impacts of DeepL Write and Claude AI on a larger sample and examine the benefit of these tools across various writing genres and disciplines. Additionally, this study did not consider individual differences in L2 proficiency or first language of the participants that might have influenced the responsiveness to the tools. The study acknowledges that the observed improvements might have been influenced by focused instruction and repeated writing practice of argumentative essays. As a result, the study findings should be interpreted as evidence of the role of these AI tools as pedagogical assistants, and not as their independent effect. Moreover, this study did not explore how the use of these AI tools might improve the critical thinking of participants, despite the close relationship between critical thinking and AW. Further research could also examine the role of teacher mediation in guiding students’ engagement with these tools. Finally, the limitations of AI tools necessitate careful scaffolding, critical digital literacy and a balance between technological aid and learners’ independence.

## Data Availability

The original contributions presented in the study are included in the article/supplementary material, further inquiries can be directed to the corresponding author/s.
